# Chloroplast Genome Analysis of Two Medicinal *Coelogyne* spp. (Orchidaceae) Shed Light on the Genetic Information, Comparative Genomics, and Species Identification

**DOI:** 10.3390/plants9101332

**Published:** 2020-10-09

**Authors:** Kai Jiang, Li-Yuan Miao, Zheng-Wei Wang, Zi-Yi Ni, Chao Hu, Xin-Hua Zeng, Wei-Chang Huang

**Affiliations:** 1 Shanghai Chenshan Plant Science Research Center, Chinese Academy of Sciences, Chenshan Botanical Garden, Shanghai 201602, China; jiangkai@csnbgsh.cn (K.J.); melodying_8829@163.com (L.-Y.M.); wangzhengwei@csnbgsh.cn (Z.-W.W.); niziyi@csnbgsh.cn (Z.-Y.N.); huchao@csnbgsh.cn (C.H.); zengxinhua@csnbgsh.cn (X.-H.Z.); 2 Shanghai Key Laboratory of Plant Functional Genomics and Resources, Shanghai Chenshan Botanical Garden, Shanghai 201602, China; 3 School of Ecological and Environmental Sciences, Shanghai Key Lab of Urban Ecological Processes and Eco-Restoration, East China Normal University, Shanghai 200241, China; 4 College of Life, Shanghai Normal University, Shanghai 200234, China; 5 College of Landscape Architecture, Fujian Agriculture and Forestry University, Fuzhou 350002, China

**Keywords:** *Coelogyne*, chloroplast genome, phylogeny, molecular identification

## Abstract

Although the medicinal properties of *Coelogyne* spp. have been previously studied, there is little genomic information providing a valuable tool for the plant taxonomy, conservation, and utilization of this genus. This study used the next-generation MiSeq sequencing platform to characterize the chloroplast (cp) genomes of *Coelogyne fimbriata* and *Coelogyne ovalis*. The Maximum Likelihood (ML) and Bayesian (BI) methods were employed to confirm the phylogenetic position of two *Coelogyne* species based on the whole chloroplast genome sequences. Additionally, we developed eight new primers based on the two cp genomes’ medium variable regions and evaluated the transferability to another 16 *Coelogyne* species. We constructed phylogenetic trees including 18 *Coelogyne* species and four outgroup species using the chloroplast fragments with the ML method. Our results showed that the cp genomes of *C. fimbriata* and *C. ovalis* contained a small single-copy region (18,839 and 18,851 bp, respectively) and a large single-copy region (87,606 and 87,759 bp, respectively), separated by two same-length inverted-repeat regions (26,675 bp in *C. fimbriata* and 26,715 bp *C. ovalis*, respectively). They all contained 86 protein-coding genes, 38 tRNA genes, and eight rRNA genes, revealing strong structure and gene content similarities. The phylogenetic analysis indicated a close relationship between the genera *Coelogyne* and *Pleione*. The newly developed primers revealed good transferability among the *Coelogyne* taxa and provided enough variable sites to distinguish *C. fimbriata* and *C. ovalis*. The two complete cp genomes and the eight new primers of *Coelogyne* provide new genomic data for further studies on phylogenomics, population genetics, and evolutionary history of *Coelogyne* taxa.

## 1. Introduction

Chloroplasts (cps) are photosynthetic organelles that play an essential role in providing energy for green plants [1]. The chloroplasts have their own genome. With a few exceptions, most chloroplast genomes consist of a single, large, circular DNA molecule, ranging in length from 120 to 160 Kb, which contains two inverted repeats (IRs) that divide the molecule into a large single-copy section (LSC) and a small single-copy section (SSC) [2]. About 100–130 genes encode about 79 proteins, 30 transfer RNAs, and four ribosomal RNAs. The cp genomes show highly conserved gene content and order [3]. Furthermore, maternal inheritance is the primary mechanism for transferring chloroplastic genetic material between generations in most angiosperms [4]. No complicated recombination events occur in the chloroplast genome. Because of its haploid nature, its high conservation in terms of gene content and order, and its simple inheritance mode, the cp genome has been employed extensively in the study of phylogeography and in addressing evolutionary questions in plants.

*Coelogyne* Lindl. (Epidendroideae; Orchidaceae) is a genus comprising more than 200 species. It is widely distributed throughout Asia, including China, India, Indonesia, and the Fiji Islands. Its main centers of diversity are in the Himalayas, Sumatra, and Borneo [5]. Most species grow in tropical montane and lowland forest areas. Some species, which grow under cooler conditions, such as *Coelogyne fimbriata* and *Coelogyne ovalis*, prefer higher altitudes on mountains. These two species are epiphytic and grow on rocks or tree trunks, with slender and creeping rhizomes. They reproduce both sexually and by vegetative growth. One or two flowers can be found on a given scape. The flowers are nectarless and attract pollinators through fragrance. According to Cheng et al.’s report in 2009, *C. fimbriata* is food-deceptive and pollinated by worker wasps [6].

A few species in this genus have been identified as medicinal plants [7,8,9]. Especially in China, India, Nepal, and Thailand, people use *Coelogyne* species as traditional medicines. For example, an alcoholic extract of pseudobulbs from *C. ovalis* contained the phenanthrenoids, coelogin, and flavidin, with these substances showing spasmolytic activity [10]. Moreover, the whole plant of *C. fimbriata* is used to reduce “heat” (primarily, inflammation) [11]. However, there are many taxonomic issues to be addressed in the genus *Coelogyne* [12]. It is still debated as to whether the two species mentioned above should be merged into one species. To better understand the phylogeny and *Coelogyne*’s species delimitations, we characterized the complete chloroplast genome sequences of *C. fimbriata* and *C. ovalis*. Using the two genomes, we developed eight primers for phylogenetic and delimited marker resources for future studies. Furthermore, we used these primers to amplify 18 *Coelogyne* species (including *C. fimbriata* and *C. ovalis*) to test the newly developed markers’ efficacy and construct a robust phylogenetic tree to improve our understanding of *Coelogyne* species’ relationship.

## 2. Results

### 2.1. Genome Sequencing and Assembly

Through the Illumina MiSeq sequencing, we obtained 3,041,719 and 3,624,370 clean reads from the *Coelogyne fimbriata* and *Coelogyne ovalis*’s total chloroplast DNA. There were 2,804,465, and 3,374,288 reads the can map to the reference genome *Calanthe sylvatica*. The results indicated similar chloroplast content and structure between the *Coelogyne* and *Calanthe* chloroplast genome. The complete cp genome sequences of *C. fimbriata* (GenBank: MK946948) and *C. ovalis* (GenBank: MK946949) were 159,795 bp and 160,040 bp in length, respectively. Based on the *C. sylvatica* reference cp genome, the four junctions between LSC/IRs and SSC/IRs of the two *Coelogyne* species were validated by PCR-based Sanger sequencing, using four pairs of primers.

### 2.2. The Organization of the Coelogyne Chloroplast Genome

**Figure 1:**
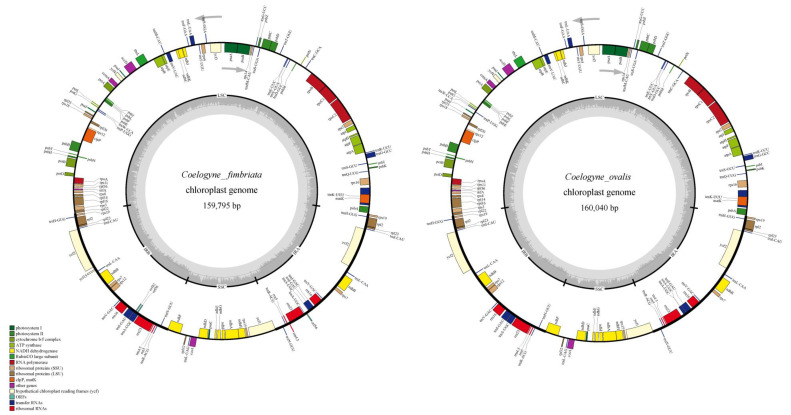
Physical maps of the complete chloroplast genomes in *Coelogyne fimbriata* and *Coelogyne ovalis*. The inner circle’s genes are transcribed in the clockwise direction, while outside genes are counterclockwise. The areas with light and dark gray coloration in the internal circle suggest guanine-cytosine (GC) content of its genome.

The chloroplast (cp) genomes of *C. fimbriata* and *C. ovalis* exhibited a typical quadripartite structure, consisting of a pair of inverted repeats (IRs) with similar length (26,7675 bp and 26,715 bp, respectively), separated by the Large single-copy (LSC) (87,606 and 87,6759 bp, respectively) and Small single-copy (SSC) (18,839 and 18,851bp, respectively) regions. The whole cp genomes of the two species, showing the guanine-cytosine (GC) contents of the LSC, SSC, and IR regions, are shown in Figure 1. In *C. fimbriata* and *C. ovalis*, GC content was very similar at 37.4% and 37.3%, respectively. However, the GC contents of the LSC and SSC regions in *C. fimbriata* (35.3% and 30.5%, respectively) and *C. ovalis* (35.2%, and 30.4%, respectively) were markedly lower than those of the IR regions (43.3% for both species).

Both cp genomes contained 86 protein-coding, 38 tRNA, and eight rRNA genes (Table 1). A total of 132 predicted functional genes were found through the annotation by DOGMA of the cp genome sequences of each of these two *Coelogyne* species. Of these, 115 genes were unique, including 81 protein-coding, 30 tRNA genes, and four rRNA genes (Figure 1, Table 2). The LSC region comprised 61 protein-coding genes and 21 tRNA genes, whereas 12 protein-coding genes and one tRNA gene were found in the SSC region. Eight protein-coding and eight tRNA genes were repeated in the IR regions. Among the 18 duplicated genes in the IR regions, six were protein-coding genes (*ndhB*, *rpl2*, *rpl23*, *rps7*, *rps19*, and *ycf2*), eight encoded tRNAs (*trnH-GUG*, *trnI-CAU*, *trnL-CAA*, *trnV-GAC*, *trnI-GAU*, *trnA-UGC*, *trnR-ACG*, and *trnN-GUU*) and four encoded rRNA (*rrn16*, *rrn5*, *rrn4.5* and *rrn23*) (Table 1). Furthermore, the number of genes with introns was 16, including ten protein-coding genes and six tRNA-coding genes (Table 2). Among them, three of these genes contained two introns: the *clpP*, *ycf3*, *rps12* genes, and a trans-spliced gene, *rps12*, with the 5′ end exon the LSC region and the intron 3′ end exon situated in the IR region (Table 3).

### 2.3. Sequence Repeats

**Figure 2:**
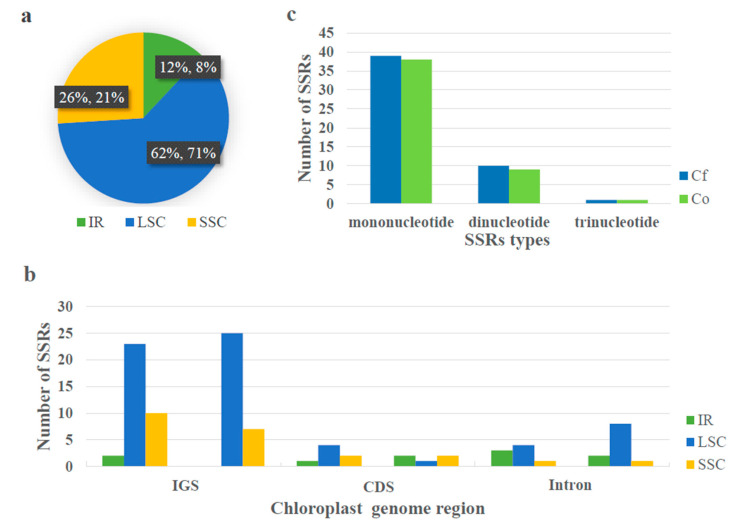
The distribution, type, and presence of simple sequence repeats (SSRs) in the chloroplast genomes of *C. fimbriata* (left) *and C. ovalis* (right). (**a**) Presence of SSRs in the regions of large single-copy region (LSC), small single-copy region (SSC) and inverted regions (IRs). (**b**) Presence of SSRs in the intergenic spacer (IGS), coding region (CDS), and Intron of LSC, SSC, and IRs regions. (**c**) Presence of the numbers of polymers.

The distribution, number, and type of microsatellites detected in the two cp genomes were analyzed. A total of 50 SSRs were found in *C. fimbriata*, of which 31 were in the LSC regions, whereas six and 13 were in the IR and SSC regions, respectively. On the other hand, in *C. ovalis*, there were 48 SSRs, with 34, four, and ten SSRs distributed in the LSC, IR, and SSC regions, respectively (Figure 2a). In addition, seven SSRs were discovered in the coding sequences (CDSs), 35 in intergenic spacers (IGSs), and eight in intron regions of the *C. fimbriata* cp genome, whereas the corresponding numbers in the *C. ovalis* cp genome were five in CDS, 32 in IGS and 11 in intron regions (Figure 2b). Among these SSRs in *C. fimbriata* and *C. ovalis*, mononucleotide repeats were the most frequent, accounting for 78% and 79%, respectively, whereas dinucleotide repeats accounted for 20% and 19%, respectively, with trinucleotide repeats accounting for 2% and 2%, respectively (Figure 2c).

Furthermore, 43 repeat sequences with different types and locations were identified in each of the two cp genomes. There were ten repeat sequences with motifs of one and ten with motifs of two in *C. fimbriata*, compared with 11 and six with motifs of one and two, respectively, in *C. ovalis*. The number of forward repeats was eight, and the number of palindrome repeats was 11, and there were no reverse or complementary repeats in *C. fimbriata*, whereas there were four, 11, and two forward, palindrome and reverse repeats, respectively, in *C. ovalis*. Of these repeats, 65% were in the same regions of the two species, with the remainder of them existing in different regions in *C. fimbriata* and *C. ovalis*.

### 2.4. Comparative Genome Analysis

**Figure 3:**
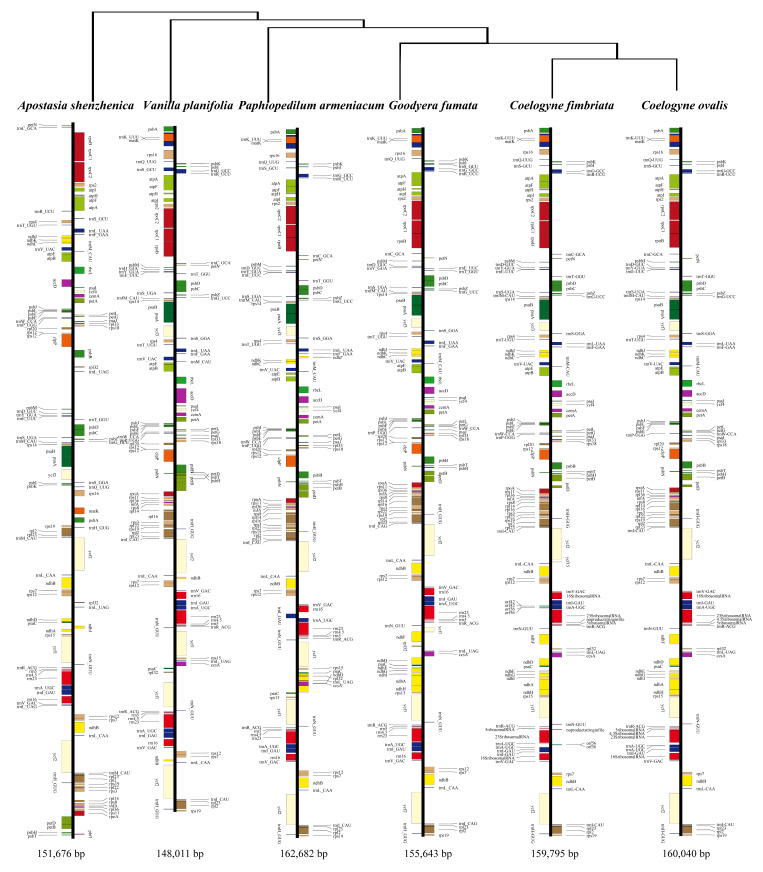
Comparative chloroplast genomes of six Orchidaceae species representing Apostasioideae (*Apostasia shenzhenica*), Vanilloideae (*Vanilla planifolia*), Cypridoideae (*Paphiopedilum armeniacum*), Orchidoideae (*Goodyera fumata*), and Epidendroideae (*Coelogyne fimbriata* and *Coelogyne ovalis*), respectively.

**Figure A1:**
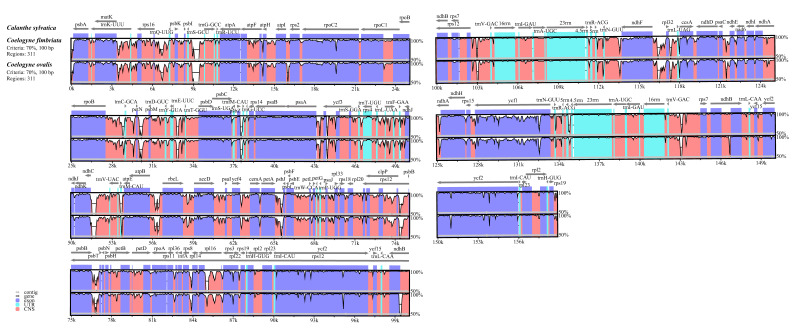
The cp genome sequence comparison of two *Coelogyne* species with *Calanthe sylvatica* as a reference. Dark grey arrows show the direction and position of genes. Pink and dark blue areas show Conserved Non-coding Sequences (CNS) and exon regions, respectively. The untranslated regions (UTRs) are colored with light-blue, including tRNA and rRNA regions. The peaks and valleys show the percent of conservation with an identity cutoff of 50%.

A total of 271 polymorphic sites can be found by comparing *C. fimbriata* and *C. ovalis* cp genomes. The nucleotide diversity (*P*_i_) was 0.0017 between the above cp genomes. According to the comparison among the six Orchidaceae species representing Apostasioideae, Vanilloideae, Cypridoideae, Orchidoideae, and Epidendroideae, we found that Apostasioideae is very different from the other Orchidaceae species in genomic structure and gene contents. However, other species except *Apostasia shenzhenica* showed similar genomic structure and gene contents (Figure 3). We chose *C. sylvatica* to be the reference genome. The mVISTA tool was used to perform the comparative analysis of cp genome sequences in three species: *C. fimbriata*, *C. ovalis*, and *C. sylvatica* (Figure A1). From the results, we could see that the IRs showed higher sequence conservation between species than did the LSC and SSC regions.

**Figure 4:**
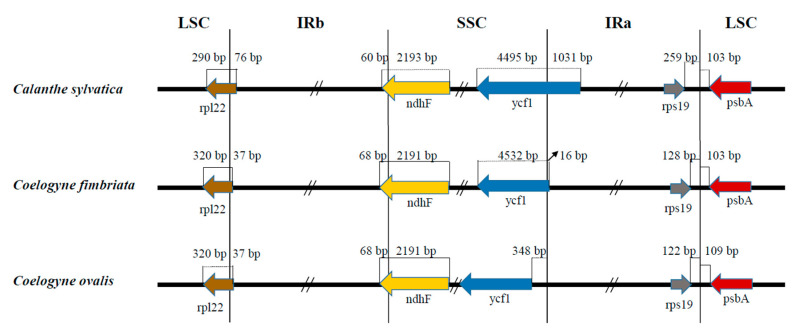
Borders comparison of the LSC, SSC, and IRs regions of two *Coelogyne* species with *C. sylvatica* as a reference. LSC: large single-copy region; SSC: small single-copy region; IRa: inverted repeat region A; IRb: inverted repeat region B.

Furthermore, the non-coding regions were revealed to be less highly conserved than the coding regions, with most of the divergences being in the IGSs. The boundary regions of these three species were also compared (Figure 4). The *rpl22* gene extended from the LSC to the inverted repeat region B (IRb) region by 76 bp in *C. sylvatica* but by 37 bp in both *C. fimbriata* and *C. ovalis*. At the boundary of IRb/SSC, the main part of the *ndhF* gene in *C. sylvatica* was in the SSC region, with 60 bp located in the IRb region, compared with 68 bp in each of the other two *Coelogyne* species. The *ycf1* gene was 1031 bp and 16 bp from the borderline between SSC and the inverted repeat region A (IRa) in *C. sylvatica* and *C. fimbriata*, respectively, whereas it was present in the SSC region in *C. ovalis*, at 348 bp from the SSC/IRa borderline. The *rps19* and *psbA* genes were distributed in the edge regions of the IRa/LSC boundary line in all three species, with the distance from these two genes, *rps19* and *psbA*, to the boundary line between IRa and LSC being 259 bp and 103 bp, respectively, in *C. sylvatica*, 128 bp and 103 bp in C. fimbriata, and 122 bp and 109 bp in *C. ovalis*. With *C. sylvatica* as the reference genome, we found that the *rpl22* gene moved away from LSC/IRb boundary line to the LSC region, whereas the *ycf1* gene shifted from the SSC/IRa boundary line to the SSC region, with genes like *ndhF* and *rps19* moving to the boundary line of IRb/SSC and IRa/LSC, respectively. Moreover, the *psbA* gene made a slight (6 bp) movement back to the LSC region in *C. ovalis*, compared with *C. fimbriata* and *C. ovalis* (Figure 4).

### 2.5. Phylogenetic Position of Coelogyne in Orchidaceae

**Figure 5:**
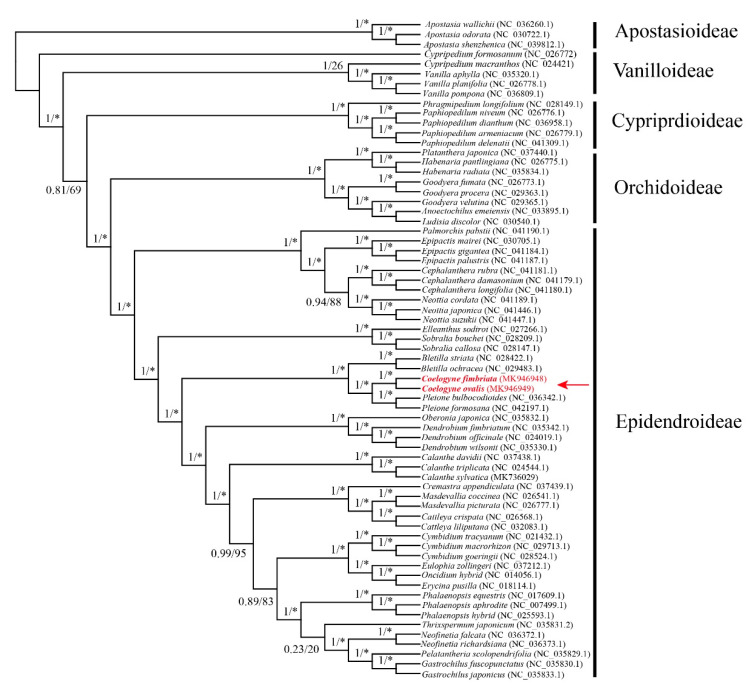
The phylogenic relationship of 67 Orchidaceae species with Maximum Likelihood (ML) and Bayesian analysis. * indicated 100 percent of bootstrap support using ML analysis.

To gain a clear insight into the phylogenetic position of *C. fimbriata* and *C. ovalis*, we carried out a phylogenetic analysis, with an aligned data matrix of the complete cp genome sequences of 67 orchid species. After removing ambiguous sites, we used 44,582 nucleotides to construct a phylogenetic tree using the Maximum Likelihood and Bayesian methods. Both results of the two methods indicated the same systematic relationship within Orchidaceae (e.g., (Vanilloideae [Orchidoideae, Epidendroideae])). It also showed the close relationship among *Pleione*, *Bletilla*, and *Coelogyne* with high bootstrap support (100) and posterior probability (1.00), which belong to the subtribe Coelogyninae Benth (Figure 5).

### 2.6. Primer Verification and Transferability

We developed eight primers based on the medium variable regions within the LSC regions to compare the whole chloroplast genomes between *C. fimbriata* and *C. ovalis.* These primers were verified in 18 species of *Coelogyne*, including *C. fimbriata* and *C. ovalis.* Most *Coelogyne* species can be amplified using the eight primers (Table A1). All the sequences which were successfully amplified have been submitted to GenBank (Table A1).

### 2.7. Phylogenetic Relationship within Coelogyne

**Figure 6:**
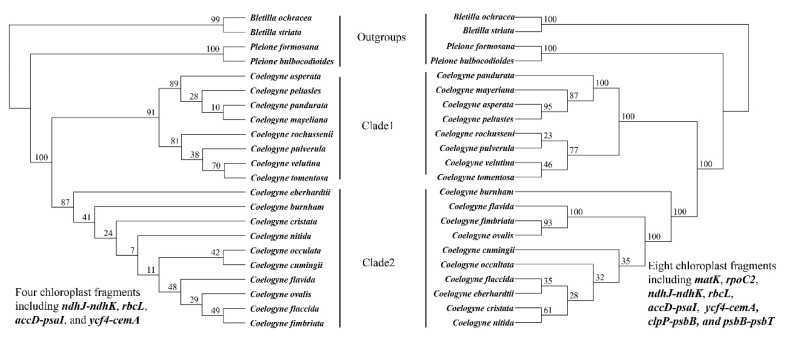
The Phylogenic relationship of 18 *Coelogyne* species with Maximum Likelihood (ML) analysis using four (left) and eight (right) chloroplast fragments, respectively. The number in the node showed the bootstrap support in the ML method using the RAxML software.

**Figure A2:**
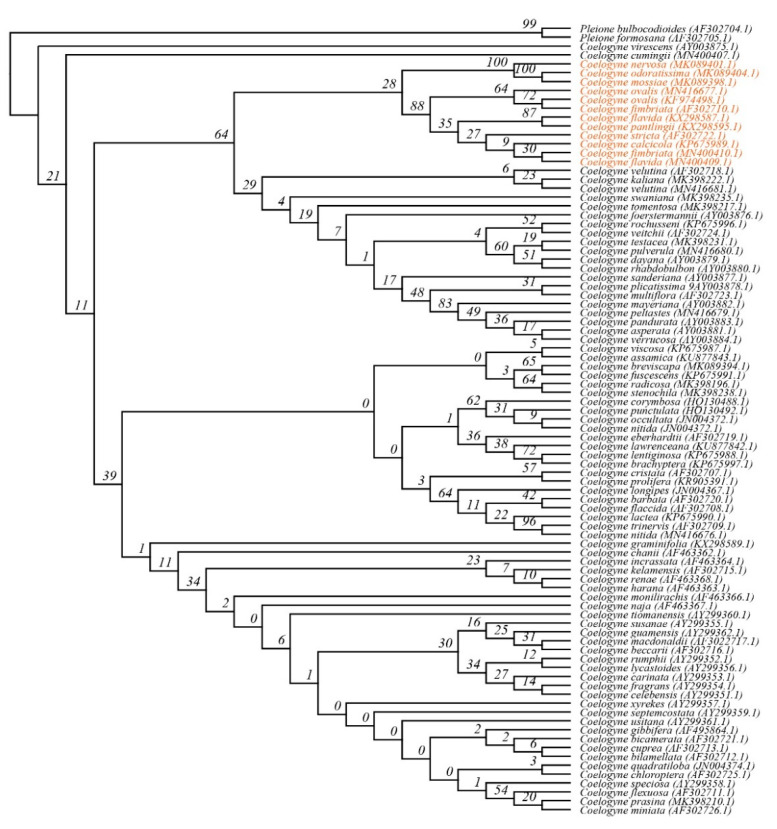
Phylogenetic tree based on 89 *matK* sequences representing 82 *Coelogyne* species using two *Pleione* species as outgroup. The number in the node showed the bootstrap support in the ML method using RAxML.

The alignments were 2858 bp and 5719 bp in the four- and eight-sequence matrix, respectively. When we considered the gap and missing data, a total of 128 and 302 polymorphic sites can be found, and the nucleotide diversity (*P*_i_) was 0.0133 and 0.0099 in the four- and eight-sequence matrix among the 18 *Coelogyne* species. There were 42 parsimony informative sites within the above two alignments. According to *Coelogyne*’s phylogenetic tree results based on four and eight fragments, two clades can be clustered with high bootstrap support (Figure 6). However, the interspecies relationship was conflicted between the two trees. Furthermore, we found that *C. fimbriata* and *C. ovalis* have the closest evolutionary relationship of all the species investigated (Figure 6). Using more *Coelogyne* species based only on *matK* sequence, a phylogenetic tree showed a low bootstrap support. In addition, the relationship between *C. fimbriata* and *C. ovalis* is still close (Figure A2).

## 3. Discussion

### 3.1. Coelogyne Chloroplast Genome Structure and Characterization

In the angiosperms, most cp genomes are ordinarily conserved with a length of 120–160 kb and a content of 100–130 genes, but some Orchidaceae species’ chloroplast genomes lost genes and rearrange structures [13]. In the current study, the cp genomes of *C. fimbriata* and *C. ovalis* each had 132 genes, consisting of 86 protein-coding genes, 38 tRNA genes, and eight rRNA genes. Moreover, the cp genome lengths of *C. fimbriata* and *C. ovalis* were 159,795 bp and 160,040 bp, respectively. This length was consistent with most angiosperms, including the Orchidaceae. There are 74 protein-coding genes shared by all angiosperms, while several other genes, such as *ycf1*, *ycf2*, *ycf4*, *rpl22*, *rpl23*, *rps16*, *ndhF*, *accD*, and *infA*, are present in only some other species [14,15,16,17,18], with variation also observed in the Orchidaceae. We found that genes with a high frequency of absence from orchid species were usually *ndhK*, *ndhF*, *ndhE*, *ndhI*, *ndhA*, *ycf15*, *ycf1*, and *psbG*, whereas genes with a low frequency of absence from orchid species were *ndhG*, *ndhD*, and *infA* [19,20,21,22]. Compared with other Epidendroideae species, the *psbG* gene was absent from the *C. fimbriata* and *C. ovalis* cp genomes [23]. The previous study showed that the *ndh* genes were present in the common ancestor of orchids but have experienced independent, significant losses at least eight times in Orchidaceae [24]. This loss may be correlated in part with the unusual life history of orchids [24]. In this study, it is unknown whether the *psbG* gene was successfully transferred to the nucleus or completely lost from the entire cell of these two species, nor was this known for the other lost genes listed above. Combined with the reason of loss in other Epidendroideae species, we speculate that this may be related to the long-term evolution of genes to adapt to extreme living environments and climatic conditions, such as high altitude for *C. ovalis* and *C. fimbriata*, which could provide us with useful information concerning the dynamics of genetic evolution.

Repeat sequences could be used to study genome recombination and rearrangement [25]. In the present study, 43 repeat sequences were detected in the cp genome of both *Coelogyne* spp. Of the four types of repetition possible, most of those in *C. fimbriata* and *C. ovalis* were palindromic (P) repeats and forward (F) repeats, with percentages of 58% and 42%, respectively, in *C. fimbriata*, and 60% and 30%, respectively, in *C. ovalis*. Repeat sequence analysis of some other orchid species takes into account only these two repeat types (P and F) regardless of the other ones (C and R) [26]. This type suggests that palindromic and forward repeats are not only typical but representative in plants. Most repeat motifs existed in the IGS regions that play an essential role in the dynamic historical analysis of plant populations [27]. Furthermore, these data will provide us with specific insights into the phylogeny and evolutionary process of these *Coelogyne* species.

SSRs are widely distributed in eukaryotic genomes, consisting of tandem repeated sequences of 1–6 nucleotide motifs as the basic repeat unit. We identified 50 SSR loci in *C. fimbriata*, among which 86% were in the non-coding regions, with 35 in the IGS and eight in intron regions. In *C. ovalis*, on the other hand, a total of 48 SSR loci were detected, among which 90% were present in the non-coding regions, with 32 in IGS and 11 in intron regions. These results indicated that most of the polymorphisms were within the IGS regions, a finding which was consistent with earlier studies showing that the cp genome repeats were often present in non-coding regions, especially in IGS regions [28,29]. These data will provide us with tremendous help in further studying genetic diversity and population structure in the Orchidaceae.

The contraction and expansion of the SSC and IR boundary regions have been regarded as mechanisms by which the length difference within the angiosperm cp genome was achieved [30]. In the current study, a comparison of IR boundaries in two *Coelogyne* species was carried out, using *C. sylvatica*, which we had sequenced before, as a reference genome (Figure A1). The results showed that those genes close to the boundary line experienced shifts to different extents, which were mainly caused by the expansion of the four regions, which, in turn, were associated with differences in genome length comparisons among these three cp genomes (Figure 4). Moreover, the length of these genes has also changed. For example, the gene of *rpl22* and *ycf1* had shortened, whereas the length of the *ndhF* gene had increased (Figure A1). According to others, this expansion and contraction usually tended to be slight and even caused the duplication of parts of or even entire genes, which usually produced pseudogenes at the boundary of IR/SSC [30]. However, this situation did not occur in the cp genomes of *C. fimbriata* and *C. ovalis*. The related data are still preliminary, and it will be necessary to obtain more information to elucidate the mechanism by which variation in gene length occurred.

### 3.2. Phylogenetic Analysis of Inter- and Intra- Coelogyne

With the rise of the high-throughput sequencing and accurate assembly technology, chloroplast genomes are inexpensive and easy to obtain [31]. Phylogenomic studies using chloroplast genomes shed light on a more innovative and profound view than single or multiple genes in the systematic evolution [30]. To construct the phylogeny tree and determine *Coelogyne*’s systematic position, we ultimately chose 67, from 28 genera, out of 122 species in the Orchidaceae, for which the full cp genome sequencing had been accomplished and officially published in the database of the NCBI. The results showed that the main relationship was the same as other studies among Vanilloideae, Orchidoideae, and Epidendroideae [32]. Within Epidendroideae, the relationship among tribes was ultimately the same as other studies using chloroplast genome CDS (coding sequence) [32]. These results showed that a systematic evolutionary relationship was robust using chloroplast genomes. Our focus genus *Coelogyne* and the *Pleione* form a high support clade (1.00 and 100 for BS and ML analysis) (Figure 5). The above clade and *Bletilla* clustered into a monophyletic tribe Arethuseae. The three genera’s systematic relationship was in line with the previous study using the restriction fragment length polymorphism (RFLP), *matK*, and *ITS* markers, but our phylogenomic tree showed higher support [12]. Based on the above analysis, we inferred the close relationship between the *Coelogyne* and *Pleione*.

Within *Coelogyne*, we used the eight chloroplast fragments to construct a phylogenetic tree, including 18 *Coelogyne* species and four outgroup species. The eight newly developed primers showed high transferability, identifying high levels of variation among *Coelogyne* (Figure A1). The results revealed two high-support clades within *Coelogyne* (Clade1 and Clade2 in Figure 6, 100 bootstrap support for ML analysis), consistent with previous studies [12]. However, the relationship among the species within each clade was different from the earlier studies [12]. On one side, there are only four shared species between ours and the previous research. It was hard to compare the different phylogenetic trees with distinct species. On the other side, most clades have high support in our analysis using the ML method. In the future, more *Coelogyne* species can be added into the phylogenetic tree using the eight chloroplast fragments, which will provide a global view of the evolutionary relationship of *Coelogyne*.

Combining NCBI data and our new sequencing *matK*, we constructed a phylogenetic tree, including 82 *Coelogyne* species. However, bootstrap support is very low in most nodes (Figure A2). The results indicated the low resolution if only one chloroplast fragment is used. More chloroplast fragments are needed to construct a robust phylogenetic tree. Chloroplast genome resources provide a potential molecular marker for the study of systematic evolution.

## 4. Materials and Methods

### 4.1. Plant Sampling and DNA Extraction

We collected fresh leaves of *Coelogyne fimbriata* and *Coelogyne ovalis* from Jiangxi and Yunnan Provinces in China, respectively (Table A2). Approximately 50 g of fresh leaves of each species were sterilized with 75% ethanol and clean with distilled water, and then these materials were stored in a 4 °C refrigerator prior to further processing. The total chloroplast genomic DNA was extracted according to the high-salt methods provided by Shi et al. 2012 [33]. Approximately 1 μg of DNA was prepared and processed to construct a DNA library according to the Illumina Sample Preparation Instructions using UltraTM DNA Library Prep Kit (New England Biolabs Inc., Ipswich, MA, USA). The cpDNA sample from each species was subjected to single-read sequencing with insertion lengths of 500 bp, using the Illumina MiSeq system (Illumina, San Diego, CA, USA). In addition, we collected leaf material of another 16 *Coelogyne* species from Shanghai Chenshan Botanical Garden (Table A2). Total DNA were extracted from the leaves using the Plant Genomic DNA Kit (TIANGEN Co., Ltd., Beijing, China).

### 4.2. Genome Assembly and Annotation

For each of the two species, low-quality reads were discarded from the raw reads, using Trimmomatic v0.39 [34] and Kmernator v1.0 software [35]. We mapped the clean reads to the reference cp genome of *Calanthe sylvatica* (GenBank accession no. MK736029) [36] with Burrows-Wheeler Aligner (BWA) v0.6 software [37]. The consensus sequences were extracted, and gaps were filled by polymerase chain reaction (PCR), with the primers designed based on the conserved sequences. According to the reference cp genome, the four LSC/IRs and SSC/IRs junctions of each of the two *Coelogyne* individuals were validated by PCR-based Sanger sequencing, using four pairs of primers. We used Dual Organellar GenoMe Annotator (DOGMA) software to initially annotate the chloroplast genomes [38]. These annotations were manually corrected for a start and stop codons and intron/exon boundaries by comparison with homologous genes in the *Calanthe sylvatica* cp genome. The tRNA genes were also verified by tRNAscan-SE v2.0 [39]. MAFFT v7.45 software [40] was employed to align the two *Coelogyne* cp genomes by comparing the structure and gene content. The online OGDRAW v1.3.1 program [41] was used to draw the two *Coelogyne* species’ circular cp genomes.

### 4.3. Repeat Sequence Analysis

Perl script MISA v2.1 [42] was used to detect microsatellites, including mono-, di-, tri-, tetra-, penta-, and hexa-nucleotide repeats. We set the thresholds at ten repeat units for mononucleotide microsatellites or simple sequence repeats (SSRs) and five repeat units for di-, tri-, tetra-, penta-, and hexa-nucleotide SSRs. The REPuter software [43] was employed to visualize forward, palindrome, reverse, and complementary sequences. The criteria of a minimum repeat size were set as 30 bp, and the sequence identity was set as higher than 90%.

### 4.4. Comparative Genome Analysis

To identify divergence hotspots within *Coelogyne* cp genomes, we conducted a sliding window analysis to evaluate the nucleotide diversity (*P*_i_) over the genomes, using DnaSP v5.10 software [44]. The window length and the step size were set to 600 and 200 bp, respectively. Genome, protein-coding gene, intron, and spacer sequence divergences were evaluated using DnaSP v5.10 [44], after alignment using MAFFT v7.45 software [40]. The chloroplast genome comparison between the two species was performed with the mVISTA program [45].

### 4.5. Phylogenetic Position of the Two Coelogyne Species

To determine the two *Coelogyne* species’ systematic position, we performed a phylogenetic analysis using the whole cp genomes. In addition to the two *Coelogyne* cp genomes, we obtained another 65 cp genome sequences, representing different lineages of Orchidaceae from the National Center for Biotechnology Information (NCBI) Organelle Genome Resource database. Three species in the genus *Apostasia* were set as the outgroups among these 67 taxa. First, we used MAFFT v.7.45 software [40] to align the 67 chloroplast genomes, setting the gap open penalty and offset value as 1.53 and 0.12, respectively. Second, Gblocks v0.91b software [46] was used to refine the alignment with allowed gap positions set as none. This software can eliminate poorly aligned positions and divergent regions. After selecting the best-fitting model of nucleotide substitution for the entire dataset (GTRGAMA) (Table A3), as determined by the Akaike Information Criterion (AIC) in MEGA X [47], the Maximum Likelihood (ML) and Bayesian (BI) analyses were performed in RAxML-HPC v8.2.11 software [48] and MrBayes v3.2 software [49], respectively. The ML analysis searches for the best trees, starting from 1000 random trees, and bootstrap percentages were obtained with 1000 non-parametric bootstrap replicates. In the BI analysis, we run the Markov chain Monte Carlo (MCMC) algorithm with two independent chains using a random starting tree and default priors for 1,000,000 generations, with trees sampled every 1000 generations. We assumed the convergence of the MCMC chains after the average standard deviation of split frequencies reached 0.01 or less. We performed ML and BI analysis on the Cyberinfrastructure for Phylogenetic Research (CIPRES) Science Gateway website v3.3 (http://www.phylo.org/).

### 4.6. Primers Design and Verification in Other Coelogyne Species

**Figure 7:**
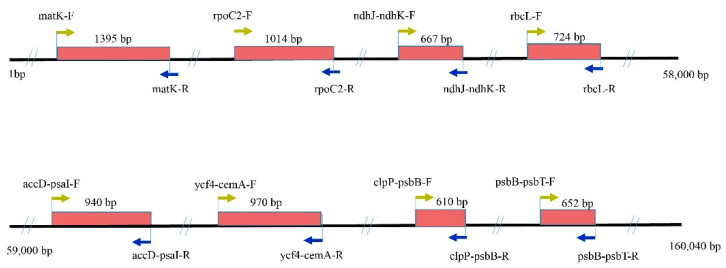
The relative positions of eight designed primers in two *Coelogyne* species. The arrow indicates the location and direction of the primer, which is amplified specifically. The rectangles in the red indicate the length of those products.

To develop more effective primers for medicinal plant identification and phylogeny analysis, we designed eight pairs of primers (Figure 7, Table 4), based on the conserved sequences on both sides of the medium variable regions within the large single-copy (LSC) regions. These primers were used to amplify and carry out Sanger sequencing of the two species and another 16 *Coelogyne* species (Table A2). First, we used these sequences to validate the two cp genomes’ accuracy by comparing eight fragments and genome sequences. Second, the efficiency of the newly developed markers was tested using these 18 *Coelogyne* species.

### 4.7. Phylogenetic Relationship within Coelogyne

To determine the 18 species’ divergence hotspot, we used DNAsp v.5.10 [44] software to calculate the number of variable sites and nucleotide diversity among the 18 species. Because some *Coelogyne* species failed to obtain all eight fragments, we created two sequence matrices. One sequence matrix includes four fragments (*ndhJ-ndhK*, *rbcL*, *accD-psaI*, and *ycf4-cemA*) shared by all 18 species, and another sequence matrix consisting of eight fragments with some missing data (Table A1). We constructed a phylogenetic tree using two sequence matrixes. We selected *Bletiall striata, Bletiall ochracea, Pleione formosana, Pleione bulbocodioides* as the outgroup species. We extracted the same sequence fragments of the eight primers’ locations after alignment with MAFFT v7.45 [40] from the whole chloroplast genome of the above four species; then, the four or eight fragments of four species were combined like all other sequences using SequenceMatrix v1.7.8 [50]. Gblocks v0.91b [46] was used to refine the alignment with allowed gap positions set as none. Phylogenetic analysis of two sequence matrices was conducted by RAxML-HPC v8.2.11 [48] using the generalised time reversible with shape parameter of the gamma distribution (GTRGAMA) model. We searched for the best trees by starting from 1000 random trees, and bootstrap percentages were obtained with 1000 non-parametric bootstrap replicates.

We also downloaded 239 *matK* sequences from the NCBI database. After removing too short and duplicate-species sequences, we obtained a total of 89 sequences (including 14 sequences from this study) and aligned these sequences representing 82 *Coelogyne* species. We chose *P. formosana* and *P. bulbocodioides* as the outgroup. After alignment using MAFFT v7.45 [40], Gblocks v0.91b [46] was used to refine the alignment with allowed gap positions set as none. Using the same parameters as the above analysis, we constructed a phylogenetic tree using RAxML-HPC v8.2.11 [48] using the GTRGAMA model. We searched for the best trees by starting from 1000 random trees, and bootstrap percentages were obtained with 1000 non-parametric bootstrap replicates.

## 5. Conclusions

To our knowledge, this was the first study to characterize the chloroplast genome of the potentially medicinal plants *C. fimbriata* and *C. ovalis*. The new cpDNA sequences will provide useful information for developing molecular markers. The results increase *Coelogyne*’s genomic data and provide fundamental references for further studies of the Coelogyneae tribe. Such genetic information can provide additional knowledge to support the conservation or the horticultural or phytopharmaceutical exploitation of these two Himalayan orchids.

## Figures and Tables

**Table 1:** Characteristics and Basic Assembly Parameters of Two *Coelogyne* Chloroplast Genomes.

Characteristics and Parameters	*C. fimbriata*	*C. ovalis*
Raw reads (bp)	3,142,569	3,763,406
Clean reads (bp)	3,041,719	3,624,370
Average read length (bp)	300	300
Number of contigs	1	1
Total length of contigs (bp)	159,795	160,040
N50 length of contigs (bp)	159,795	160,040
Total cp genome size (bp)	159,795	160,040
LSC length (bp)	87,606	87,759
SSC length (bp)	18,839	18,851
IR length (bp)	26,675	26,715
Total CDS length (bp)	79,891	78,258
Total tRNA length (bp)	2865	2911
Total rRNA length (bp)	9038	9041
Total GC content (%)	37.39	37.35
GC content for LSC (%)	35.30	35.20
GC content for SSC (%)	30.50	30.40
GC content for IR (%)	43.30	43.30
Total number of genes	136	133
Protein-coding genes	90	87
rRNAs genes	38	38
tRNAs genes	8	8
Duplicated genes	17	17

Note, cp: Chloroplast; LSC: large single-copy region; SSC: small single-copy region; IR: inverted region; CDS: coding region; GC: guanine-cytosine.

**Table 2:** Gene Composition of the *Coelogyne* Chloroplast Genome.

Categories of Genes	Groups of Genes	Name of Genes
RNA genes	Ribosomal RNAs	*rrn5*^a^, *rrn4.5*^a^, *rrn16*^a^, *rrn23*^a^
	Transfer RNAs	*trnK-UUU*^b^, *trnQ-UUG*, *trnS-GCU*, *trnG-GCC*^b^, *trnR-UCU*, *trnC-GCA*, *trnD-GUC*, *trnA-UGC*, *trnY-GUA*, *trnE-UUC*, *trnF-GAA*, *trnT-GGU*, *trnS-UGA*, *trnG-UCC*, *trnfM-CAU*, *trnS-GGA*, *trnT-UGU*, *trnL-UAA*^b^, *trnF-GAA*, *trnV-UAC*^b^, *trnM-CAU*, *trnW-CCA*, *trnP-UGG*, *trnH-GUG*^a^, *trnI-CAU*^a^, *trnL-CAA*^a^, *trnV-GAC*^a^, *trnI-GAU*^a,b^, *trnA-UGC*^a,b^, *trnR-ACG*^a^, *trnN-GUU*^a^, *trnL-UAG*, *trnS-GCU*
Transcription- and translation-related genes	Small subunit of ribosome	*rps2*, *rps3*, *rps4*, *rps7*^a^, *rps8*, *rps11*, *rps12*^c^, *rps14*, *rps15*, *rps16*^b^, *rps18*, *rps19*^a^
	Large subunit of ribosome	*rpl2*^a,b^, *rpl14*, *rpl16*^b^, *rpl20*, *rpl22*, *rpl23*^a^, *rpl32*, *rpl33*, *rpl36*
	Transcription	*rpoA*, *rpoB*, *rpoC1*^b^, *rpoC2*
	Translation initiation factor	*infA*
Photosynthesis-related genes	NADH dehydrogenase	*ndhA*^b^, *ndhB*^a,b^, *ndhC*, *ndhD*, *ndhE*, *ndhF*, *ndhG*, *ndhH*, *ndhI*, *ndhJ*, *ndhK*
	Photosystem I	*psaA*, *psaB*, *psaC*, *psaI*, *psaJ*
	Photosystem II	*psbA*, *psbB*, *psbC*, *psbD*, *psbE*, *psbF*, *psbH*, *psbI*, *psbK*, *psbL*, *psbJ*, *psbN*, *psbT*, *psbZ*, *psbM*
	RubisCO large subunit	*rbcL*
	Cytochrome b/f complex	*petA*, *petB*^b^, *petD*, *petG*, *petL*, *petN*
	ATP synthase	*atpA*, *atpB*, *atpE*, *atpF*^b^, *atpH*, *atpI*
	Cytochrome c synthesis	*ccsA*
Others	RNA processing	*matK*
	Carbon metabolism	*cemA*
	Fatty acid synthesis	*accD*
	Proteolysis	*clpP* ^c^
Genes of unknown function	Conserved reading frames	*ycf1*, *ycf2*^a^, *ycf4*, *ycf3*^c^, *ycf15*, *ycf68*^d^

^a^ Gene with two copies; ^b^ Gene with one intron; ^c^ Gene with two introns. ^d^ Gene existed in which species chloroplast genome and copy number and intron number in each chloroplast (cp) genome. NADH: Nicotinamide adenine dinucleotide.

**Table 3:** Location and Length of Intron-Containing Genes in the *Coelogyne* Chloroplast Genome.

Gene	Location	Nucleotides in Base Pairs				
		Exon I	Intron I	Exon II	Intron II	Exon III
*atpF*	LSC	144/144	965/964	411/411		
*clpP*	LSC	69/69	963/950	291/291	675/673	252/252
*ndhA*	SSC	552/552	1235/1235	540/540		
*ndhB*	IR	777/777	701/710	756/756		
*petB*	LSC	6/6	739/736	642/642		
*rpl16*	LSC	9/9	1007/1248	399/399		
*rpl2*	IR	387/387	663/663	432/432		
*rpoC1*	LSC	435/435	766/778	1617/1617		
*rps12* ^a^	LSC	126/126	-	232/232	549/549	26/26
*rps16*	LSC	40/40	894/893	248/248		
*ycf3*	LSC	126/126	721/723	228/228	672/672	152/152
*trnG-GCC*	LSC	23/23	700/700	47/47		
*trnI-GAU*	IR	42/42	948/948	35/35		
*trnK-UUU*	LSC	37/37	2915/2917	26/26		
*trnL-UAA*	LSC	35/35	574/574	50/50		
*trnV-UAC*	LSC	39/39	577/577	35/35		

^a^ The *rps12* is a trans-spliced gene with the 5′ end located in the LSC region and duplicated in the 3′ end in the IR regions. LSC: large single-copy region; SSC: small single-copy region; IR: inverted repeat region.

**Table 4:** Basic Information of Eight Chloroplast Primers.

Locus	Primer Sequence (5′-3′)	Location	Product Length (bp)	Annealing Temperature/Tm (°C)
*matK*	F: CACCAGATCATTGATACGGA	CDS	1395	55
	R: CCTGTGGAAATTCTCGGTTA			
*rpoC2*	F: TATTGTCCATGCCTCTTCAC	CDS	1014	55
	R: CATTTTTCTGGAGAGGTGGA			
*ndhJ-ndhK*	F: CCTATCCAACTTTCAGGCAT	IGS	667	55
	R: ATCACAAGTTTGACCTTCGA			
*rbcL*	F: TCGAGTAGACCTTGTTGTTG	IGS	724	55
	R: CGGCACAAAATAAGAAACGA			
*accD-psaI*	F: TGTTTTCTTTGGGGACATCA	IGS	940	55
	R: CGGAAAGGCCACATATCATA			
*ycf4-cemA*	F: TGAGAATTTGACTCCACGAG	IGS	970	55
	R: ATTTCGGATTGCCTGGTATT			
*clpP-psbB*	F: ACACCAATGGGCATTAAGAT	IGS	610	55
	R: ACCTGTTCGGTAGATTTTGT			
*psbB-psbN*	F: ATGCTCAAGTGGAATTTGGA	IGS	652	55
	R: GAACTTTAGGTGGTTCTCGA			

CDS: coding region; IGS: intergenic spacer.

## Appendix A

**Table A1:** GenBank Accession of Eight Chloroplast Fragments for 18 *Coelogyne* Species in the Study.

Species	GenBank Accession							
	*matK*	*rpoC2*	*ndhJ-ndhK*	*rbcL*	*accD-psaI*	*ycf4-cemA*	*clpP-psbB*	*psbB-psbT*
*C. rochussenii*	-	-	MN512535	MN416673	MN512468	MN512517	MN512484	-
*C. burnham*	MN400405	MN400397	MN512520	MN396950	MN512453	MN512502	MN512471	MN512487
*C. veluting*	MN416681	MN416666	MN512537	MN416675	MN512470	MN512519	MN512486	MN512501
*C. mayeliana*	MN400412	MN400404	MN512528	MN400420	MN512461	MN512510	-	-
*C. peltasles*	MN416679		MN512532	MN416670	MN512465	MN512514	MN512482	MN512498
*C. cumingii*	MN400407	MN400399	MN512522	MN400414	MN512455	MN512504	MN512473	MN512489
*C. flavida*	MN400409	MN400401	MN512525	MN400417	MN512458	MN512507	MN512476	MN512492
*C. eberhardtii*	MN400408	MN400400	MN512524	MN400416	MN512457	MN512506	MN512475	MN512491
*C. cristata*	-	-	MN512523	MN400415	MN512456	MN512505	MN512474	MN512490
*C. tomentosa*	-	MN416665	MN512536	MN416674	MN512469	MN512518	MN512485	MN512500
*C. occulata*	MN416678	MN416661	MN512531	MN416669	MN512464	MN512513	MN512481	MN512497
*C. flaccida*	MN400411	MN400403	MN512527	MN400419	MN512460	MN512509	MN512478	MN512494
*C. pulverula*	MN416680	MN416663	MN512534	MN416672	MN512467	MN512516	MN512483	MN512499
*C. asperata*	MN400406	MN400398	MN512521	MN400413	MN512454	MN512503	MN512472	MN512488
*C. pandurata*	-	MN416662	MN512533	MN416671	MN512466	MN512515	-	-
*C. nitida*	MN416676	MN416659	MN512529	MN416667	MN512462	MN512511	MN512479	MN512495
*C. fimbriata*	MN400410	MN400402	MN512526	MN400418	MN512459	MN512508	MN512477	MN512493
*C. ovalis*	MN416677	MN416660	MN512530	MN416668	MN512463	MN512512	MN512480	MN512496

- indicate the failed PCR.

**Table A2:** Specimen Information for the *Coelogyne* Spp. Samples Used in This Study.

Species	Collector	Collection No.	Deposited Institution	n
*C. fimbriata*	Wei-Chang Huang	CS-HWC201606-2	CSH	1
*C. ovalis*	Wei-Chang Huang	CS-HWC201606-5	CSH	1
*C. rochussenii*	Kai Jiang	CS-JK201806-01	CSH	1
*C. burnham*	Kai Jiang	CS-JK201806-02	CSH	1
*C. veluting*	Kai Jiang	CS-JK201806-03	CSH	1
*C. mayeliana*	Kai Jiang	CS-JK201806-04	CSH	1
*C. peltasles*	Kai Jiang	CS-JK201806-05	CSH	1
*C. cumingii*	Kai Jiang	CS-JK201806-06	CSH	1
*C. flavida*	Kai Jiang	CS-JK201806-07	CSH	1
*C. eberhardtii*	Kai Jiang	CS-JK201806-08	CSH	1
*C. cristata*	Kai Jiang	CS-JK201806-09	CSH	1
*C. tomentosa*	Kai Jiang	CS-JK201806-10	CSH	1
*C. occulata*	Kai Jiang	CS-JK201806-11	CSH	1
*C. flaccida*	Kai Jiang	CS-JK201806-12	CSH	1
*C. pulverula*	Kai Jiang	CS-JK201806-13	CSH	1
*C. asperata*	Kai Jiang	CS-JK201806-14	CSH	1
*C. pandurata*	Kai Jiang	CS-JK201806-15	CSH	1
*C. nitida*	Kai Jiang	CS-JK201806-16	CSH	1

All voucher specimens were deposited in shanghai chenshan herbarium (CSH), shanghai, China. all the materials were collected in living plants from Shanghai Chenshan Botanical Garden. n showed the number of collected sample.

**Table A3:** Best model selection based on the Maximum Likelihood method.

Model	Param	BIC	AICc	lnL	Invariant	Gamma	R
GTR + G	140.00	582,962.94	581,155.75	−290,437.87	n/a	0.94	1.39
GTR + G + I	141.00	582,977.87	581,157.77	−290,437.88	0.00	0.94	1.39
T92 + G	134.00	585,265.34	583,535.60	−291,633.79	n/a	0.93	1.48
TN93 + G	137.00	585,287.36	583,518.89	−291,622.44	n/a	0.93	1.48
HKY + G	136.00	585,287.51	583,531.95	−291,629.97	n/a	0.93	1.48
T92 + G + I	135.00	585,403.95	583,661.30	−291,695.64	0.00	0.93	1.57
TN93 + G + I	138.00	585,425.88	583,644.51	−291,684.25	0.00	0.92	1.57
HKY + G + I	137.00	585,426.37	583,657.91	−291,691.95	0.00	0.93	1.57
GTR + I	140.00	587,343.22	585,536.03	−292,628.01	0.31	n/a	1.37
T92 + I	134.00	589,665.50	587,935.76	−293,833.87	0.31	n/a	1.34
HKY + I	136.00	589,688.10	587,932.55	−293,830.27	0.31	n/a	1.34
TN93 + I	137.00	589,690.94	587,922.48	−293,824.23	0.31	n/a	1.34
K2 + G	133.00	591,575.20	589,858.37	−294,796.18	n/a	0.84	1.56
K2 + G + I	134.00	591,756.46	590,026.72	−294,879.35	0.00	0.83	1.65
GTR	139.00	592,361.70	590,567.41	−295,144.70	n/a	n/a	1.33
T92	133.00	594,707.63	592,990.80	−296,362.39	n/a	n/a	1.32
HKY	135.00	594,729.93	592,987.28	−296,358.63	n/a	n/a	1.32
TN93	136.00	594,733.26	592,977.71	−296,352.85	n/a	n/a	1.32
K2 + I	133.00	596,345.39	594,628.56	−297,181.27	0.33	n/a	1.45
JC + G	132.00	600,630.85	598,926.93	−299,331.46	n/a	0.87	0.50
JC + G + I	133.00	600,645.77	598,928.94	−299,331.46	0.00	0.87	0.50
K2	132.00	602,173.44	600,469.52	−300,102.75	n/a	n/a	1.39
JC + I	132.00	605,419.13	603,715.21	−301,725.60	0.32	n/a	0.50
JC	131.00	610,996.30	609,305.29	−304,521.64	n/a	n/a	0.50

Models with the lowest Bayesian Information Criterion (BIC scores) are considered to describe the substitution pattern the best. For each model, the Akaike Information Criterion, corrected (AICc) value, Maximum Likelihood value (lnL), and the number of parameters (including branch lengths) are also presented. Non-uniformity of evolutionary rates among sites may be modeled by using a discrete Gamma distribution (+G) with five rate categories and by assuming that a certain fraction of sites is evolutionarily invariable (+I). Whenever applicable, estimates of the gamma shape parameter and the estimated fraction of invariant sites are shown. Assumed or estimated values of transition/transversion bias (R) are shown for each model, as well. For estimating ML values, a tree topology was automatically computed. This analysis involved 67 nucleotide sequences. There were a total of 44,582 positions in the final dataset. Evolutionary analyses were conducted in MEGA X.
